# Supplementary material for: Gastric Cancer in Northern Canadian Populations: A Focus on Cardia and Non-Cardia Subsites
Source: Cancers (Basel). 2019 Apr 15;11(4):534. doi: 10.3390/cancers11040534 (PMC6520747; doi:10.3390/cancers11040534)
Supplement: Supplementary file 1 [file cancers-11-00534-s001.pdf]

Supplementary Materials: Gastric Cancer in Northern Canadian Populations: A Focus on Cardia and Non-Cardia Subsites

Amy Colquhoun, Heather Hannah, André Corriveau, Brendan Hanley, Yan Yuan, Karen J. Goodman and The CAN*Help* Working Group

Table S1. Comparison of the number of cases and estimated standardized incidence ratios (SIRs) using two different definitions of non-cardia gastric cancer by population and sex; Northwest Territories and Yukon; various years.

Population	Non-Cardia Gastric Cancer	Men				Women			
		Cases [1]	SIR [2]	95% CI [3]		Cases [1]	SIR [2]	95% CI [3]	
Yukon	C16.1-9	14	1.3	0.71	2.2	9	1.5	0.71	2.9
	C16.1-4	7	1.6	0.62	3.2	3	1.3	0.26	3.6
NT - non-Indigenous	C16.1-9	9	0.94	0.43	1.8	3	0.62	0.13	1.8
	C16.1-4	3	0.75	0.15	2.2	0	0	0.00	1.9
NT - Indigenous	C16.1-9	25	2.7	1.7	3.9	18	3.1	1.8	4.8
	C16.1-4	14	3.6	2.0	6.0	12	4.9	2.5	8.6

SIR, standardized incidence ratio; CI, confidence interval; NT, Northwest Territories; [1] Yukon includes cases diagnosed during 2003–2012; NT includes cases diagnosed during 1997–2015; [2] SIRs compare study population to Canada during 2003–2007; [3] 95% CIs are based on the Poisson distribution.

Table S2. Comparison of the number of cases and estimated age-standardized incidence rates (ASRs per 100,000) using two different definitions of non-cardia gastric cancer by population and sex, northern territories and Canada; various years.

Population	Non-Cardia Gastric Cancer	Men				Women			
		Cases [1]	ASR [2]	95% CI [3]		Cases [1]	ASR [2]	95% CI [3]	
Canada	C16.1-9	6489	5.0	4.9	5.1	4544	2.8	2.7	2.9
	C16.1-4	2702	2.1	2.0	2.2	1884	1.1	1.0	1.2
Yukon	C16.1-9	6	6.1	1.1	11.1	4	4.5	0.0	9.1
	C16.1-4	1	0.3	0.0	1.7	2	0.8	0.0	3.0
NT - non-Indigenous	C16.1-9	9	5.8	1.5	10.1	3	2.0	0.0	4.3
	C16.1-4	3	1.7	0.0	3.7	0	0	0.0	0.0
NT - Indigenous	C16.1-9	25	13.3	8.0	18.6	18	7.7	4.1	11.3
	C16.1-4	14	7.8	3.7	11.9	12	5.2	2.2	8.2

ASR, age-standardized incidence rate; CI, confidence interval; NT, Northwest Territories; [1] Canada and Yukon include cases diagnosed during 2003–2007; NT includes cases diagnosed during 1997–2015; [2] ASRs standardized to the World Population; [3] 95% CIs are based on the Poisson distribution.

Table S3. Estimated gastric cancer age-standardized incidence rates (ASRs per 100,000) by subsite, population, and sex using two different standard populations; northern territories and Canada; various years.

Population	Gastric Cancer Subsite [1]	Men				Women			
		Cases [2]	ASR	95% CI [3]		Cases [2]	ASR	95% CI [3]	
Standard population: World (Segi)									
Canada	All	16,872	7.0	6.9	7.1	9510	3.2	3.1	3.3
	CGC	3151	2.5	2.4	2.6	857	0.6	0.6	0.6
	NCGC	6489	5.0	4.9	5.1	4544	2.8	2.7	2.9
Yukon	All	21	9.2	5.2	13.2	12	5.8	2.4	9.2
	CGC	3	3.3	0.0	7.2	1	0.9	0.0	2.7
	NCGC	6	6.1	1.1	11.1	4	4.5	0.0	9.1
NT - non-Indigenous	All	16	8.8	3.9	13.7	3	2.0	0.0	4.3
	CGC	7	3.0	0.6	5.4	0	0.0	0.0	0.0
	NCGC	9	5.8	1.5	10.1	3	2.0	0.0	4.3
NT - Indigenous	All	26	13.8	8.4	19.2	18	7.7	4.1	11.2
	CGC	1	0.5	0.0	1.4	0	0.0	0.0	0.0
	NCGC	25	13.3	8.0	18.6	18	7.7	4.1	11.3
Standard population: Canada 2011									
Canada	All	16,872	4.2	3.9	4.4	9510	6.4	6.2	6.6
	CGC	3151	4.8	4.6	5.0	857	1.1	1.0	1.2
	NCGC	6489	10.4	10.1	10.7	4544	5.6	5.4	5.8
Yukon	All	21	16.4	9.0	23.8	12	9.3	3.7	14.9
	CGC	3	5.7	0.0	12.4	1	3.6	0.0	10.7
	NCGC	6	11.1	1.7	20.5	4	5.6	0.0	11.4
NT - non-Indigenous	All	16	20.6	6.5	34.7	3	5.0	0.0	11.1
	CGC	7	5.6	0.7	10.5	0	0.0	0.0	0.0
	NCGC	9	15.1	1.9	28.3	3	5.0	0.0	11.1
NT - Indigenous	All	26	25.3	14.7	35.9	18	14.8	7.5	22.1
	CGC	1	0.6	0.0	1.9	0	0.0	0.0	0.0
	NCGC	25	24.6	14.1	35.1	18	14.8	7.5	22.1

ASR, age-standardized incidence rate; CI, confidence interval; CGC, cardia gastric cancer; NCGC, non-cardia gastric cancer; NT, Northwest Territories; [1] CGC defined as C16.0; NCGC defined as C16.1-9; [2] For Canada and Yukon, all gastric cancer cases combined include cases diagnosed during 2003–2012; gastric cancer subsites include cases diagnosed during 2003–2007. NT includes cases diagnosed during 1997–2015. [3] 95% CIs are based on the Poisson distribution.

© 2019 by the authors. Licensee MDPI, Basel, Switzerland. This article is an open access article distributed under the terms and conditions of the Creative Commons Attribution (CC BY) license (http://creativecommons.org/licenses/by/4.0/).
